# Use of near-infrared spectroscopy during a vascular occlusion test to assess the microcirculatory response during fluid challenge

**DOI:** 10.1186/cc10449

**Published:** 2011-09-16

**Authors:** Emmanuel Futier, Sebastien Christophe, Emmanuel Robin, Antoine Petit, Bruno Pereira, Jacques Desbordes, Jean-Etienne Bazin, Benoit Vallet

**Affiliations:** 1 Department of Anaesthesiology and Critical Care Medicine, Estaing Hospital, University Hospital of Clermont-Ferrand, 1 place Lucie Aubrac, F-63000, Clermont-Ferrand, France; 2 Department of Anaesthesiology and Critical Care Medicine, University Hospital of Lille, University Nord de France, Rue du Pr. Emile Laine, F-59037, Lille, France; 3 Department of Clinical Research (DRCI), Biostatics Unit, University Hospital of Clermont-Ferrand, place Henri Dunant, F-63000, Clermont-Ferrand, France; 4 Federation of Anaesthesiology and Critical Care Medicine, University Hospital of Lille, University Nord de France, Rue du Pr. Emile Laine, F-59037, Lille, France

## Abstract

**Introduction:**

Adequate volume expansion (VE) in patients with evidence of hypoperfusion should be aimed not only at achieving an increase in stroke volume (SV) and cardiac index (CI) but also at improved tissue perfusion and oxygenation. Our aim in this study was to assess the dynamic changes in muscle tissue oxygen saturation (StO_2_) during hypovolaemia and in response to VE.

**Methods:**

We conducted a prospective study of 42 fluid challenges in patients undergoing major abdominal surgery with evidence of hypovolaemia, defined as pulse pressure variation (PPV) >13% and SV variation (SVV) >12%. CI, SV, SVV (FloTrac/Vigileo) and PPV were measured before and after VE. Fluid responsiveness was defined as an increase of SV >15% after a 500-mL colloid infusion over 15 minutes. In all patients, the muscle StO_2 _and its changes during a standardised vascular occlusion test were analysed using a near-infrared spectroscopy device after anaesthesia induction (which defined the baseline state) and before and after each VE.

**Results:**

No patients were preload-responsive after anaesthesia induction. Twenty-nine of forty-two fluid challenges (69%) were positive for VE, with a statistically significant (*P *< 0.001) difference in SV changes between positive and negative responses to VE. There was a statistically significant difference in PPV and SVV values before VE in the positive and negative fluid responses [PPV: 16% (15% to 18%) vs. 14% (13% to 15%), *P *= 0.001; and SVV: 14% (13% to 16%) vs. 16% (15% to 16%), *P *= 0.03 or positive and negative fluid responses, respectively]. Data are presented as medians and 25th and 75th percentiles Before VE there was no significant difference in StO_2 _values relative to baseline [86% (78% to 88%) vs. 84% (77% to 91%), *P *= 0.83], without a significant difference (*P *= 0.36) between positive and negative fluid challenges. Hypovolaemia was associated with a significant reduction (*P *= 0.004) in StO_2 _recovery slope, with a significant difference (*P *= 0.02) between positive and negative fluid challenges. The VE-induced increase in the StO_2 _recovery slope was 62 ± 49% (*P *< 0.001) for positive fluid challenges and 26 ± 34% (*P *= 0.04) for negative fluid challenges.

**Conclusions:**

Hypovolaemia significantly affects the muscle StO_2 _recovery slope. Restoring effective intravascular volume with fluid loading significantly improves the StO_2 _recovery slope, despite apparently ineffective changes in systemic haemodynamics.

## Introduction

Fluid loading is a first-line therapy when hypovolaemia is suspected in patients with evidence of hypoperfusion, and it is commonly used in operating rooms and ICUs. The maintenance of adequate oxygen delivery and tissue perfusion is considered a primary goal in volume replacement [1] while avoiding fluid overload, which may lead to interstitial oedema [2]. Several studies have demonstrated the superiority of dynamic preload indices, such as pulse pressure variation (PPV) and stroke volume variation (SVV), rather than static indices for individualised evaluation of patients who are likely to benefit from an increase in preloading [3-5]. In addition, the use of SVV or PPV can reduce organ failure during individualised, goal-directed fluid optimisation [6,7].

Although a fluid challenge should correct macrohaemodynamics (stroke volume (SV) and cardiac output (CO)), the ideal volume replacement strategy should also improve microcirculation perfusion and tissue oxygenation. Hypovolaemia during major surgery or sepsis leads to inadequate perfusion of the microcirculation and insufficient oxygen availability to meet tissue oxygen needs [8]. However, previous reports have suggested a mismatch between global haemodynamics and microcirculation and a potential independence of macrocirculation and microcirculation during fluid loading [9,10]. Thus, fluid administration may correct systemic haemodynamic variables but not regional and microcirculatory oxygenation and perfusion [11].

Microcirculatory haemoglobin and oxygen availability can be measured by use of near-infrared spectroscopy (NIRS) [12], a noninvasive technique that can be performed at the bedside. In this method, the differential absorption of infrared light at two specific wavelengths (680 and 800 nm) by deoxyhaemoglobin is used to define the haemoglobin saturation level in vessels located in the tissue volume that is illuminated by the probe [13]. The dynamic response to tissue oxygen saturation (StO_2_), especially the StO_2 _recovery slope, during a standardised vascular occlusion test (VOT) is assumed to reflect the recruitment of microvessels in response to a local hypoxic stimulus [14]. Researchers in previous studies found that the StO_2 _recovery slope was a prognostic factor in septic patients [15] and was useful in evaluating the response to norepinephrine in severely hypotensive septic shock patients [16]. However, there is no information on the StO_2 _response during fluid resuscitation and in the presence of abnormal vascular reactivity in patients undergoing major surgery. Our aim in this study was to assess thenar muscle StO_2 _changes during VOT in responses to hypovolaemia and to assess the dynamic responses of the StO_2 _recovery slope in response to volume expansion (VE).

## Materials and methods

This study was approved by the Ethics Committee of our institution. The requirement for written consent was waived, as no interventions were required. The protocol was part of our routine practise in patients undergoing major abdominal surgery. All patients had arterial catheters for invasive blood pressure monitoring. The haemodynamic measurements and fluid loading are routinely used to assess fluid responsiveness.

We studied 24 consecutive Caucasian patients (13 males and 11 females), all with American Society of Anesthesiology Physical Status scores of 2 or 3, who were undergoing major abdominal surgery. Patients with permanent cardiac arrhythmia, aortic regurgitation, body mass index ≥35 kg/m^2^, those receiving β-blocker therapy and those with contraindications for VOT (arteriovenous shunt) were excluded. The surgical procedures that our 24 patients underwent were duodenopancreatectomy (*n *= 9), colectomy (*n *= 10), gastrectomy (*n *= 3) and hepatectomy (*n *= 2), and all were scheduled for tumour resection.

### Study design

Standardised anaesthetic management was applied for all patients. General anaesthesia was induced with propofol (2 to 3 mg/kg), sufentanil (0.2 to 0.3 μg/kg) and cisatracurium (0.15 mg/kg) to facilitate endotracheal intubation and was maintained with a continuous infusion of propofol and sufentanil (using target-controlled infusion) to target a bispectral index of 40 to 50 (Aspect A-1000; Aspect Medical Systems, Norwood, MA, USA). Anaesthetic concentrations were based on predicted body weight. After tracheal intubation all patients were ventilated in the supine position in controlled volume mode using a tidal volume of 8 to 10 mL/kg of predicted body weight, a respiratory rate adjusted to maintain an end-tidal carbon dioxide tension of 30 to 35 mmHg, an inspiratory/expiratory ratio of 1:2 and a positive end-expiratory pressure of 5 cmH_2_O. The inspiratory oxygen fraction was set at 0.5 (Datex-Ohmeda Avance; GE Healthcare, Helsinki, Finland). Ventilatory settings were kept constant during the entire study period. Intraoperative fluid intake was maintained using 8 mL/kg/hour of lactated Ringer's solution. Normothermia was maintained during the entire procedure using a convective air warming system (WarmTouch; Tyco Healthcare, Pleasanton, CA, USA).

### Measurements

Standard monitoring included measurements with a five-lead continuous electrocardiograph and measurements of heart rate, peripheral oxygen saturation and end-tidal partial carbon dioxide tension. As part of our routine haemodynamic monitoring during major surgery, all patients intubated with a 20-gauge, 8-cm arterial catheter (Arrow International, Reading, PA, USA), which was inserted into the left radial artery. Arterial pressure was measured using a high-fidelity dedicated pressure transducer (FloTrac Sensor; Edwards Lifesciences, Irvine, CA, USA) connected to a Vigileo version 3.01 monitor (Edwards Lifesciences) and a bedside monitor (IntelliVue MP50; Philips Medical Systems, Suresnes, France). The pressure transducer was levelled at the midaxillary line, zeroed at atmospheric pressure and fixed to the operating table so that the transducer was at the level of the atrium during the study protocol. In all patients, automated online PPV and SVV were measured continuously from the algorithm integrated in the monitors as described in detail elsewhere [5,17,18]. The following variables were recorded before and after each episode of VE: cardiac index (CI), SV, systolic arterial blood pressure, mean arterial blood pressure (MAP) and diastolic arterial blood pressure.

### Near-infrared spectroscopy and vascular occlusion test

The StO_2 _was continuously and noninvasively measured using the InSpectra™ StO_2 _System (model 650; Hutchinson Technology Inc., Hutchinson, MN, USA). A 15-mm NIRS sensor probe (model 1615; Hutchinson Technology Inc.) placed on the right thenar eminence allowed us to measure StO_2 _at a depth of 14 mm. StO_2 _values were recorded continuously and stored every two seconds by the NIRS monitor. StO_2 _stability was defined as variation <2% over 30 seconds (pre-VOT StO_2_) [19]. The values were then transferred to a personal computer and analysed using a dedicated program (InSpectra Analysis Program version 4.0; Hutchinson Technology Inc.).

**Figure 1:**
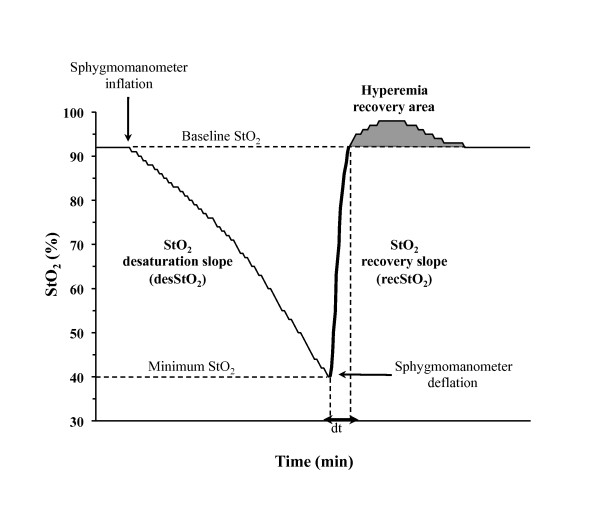
**Response to tissue oxygen saturation during a vascular occlusion test**. StO_2_, tissue oxygen saturation. dt = time to reach the StO_2 _baseline (preVOT) value. The two-way arrow is dt.

The VOT was performed by using a sphygmomanometer placed around the upper arm. The sphygmomanometer was rapidly inflated to 50 mmHg more than systolic pressure and was kept inflated until StO_2 _decreased to 40% [16]. Upon the completion of the ischaemic period, the sphygmomanometer was rapidly deflated and the StO_2 _response was followed until it returned to the baseline value. For every test, the following VOT-derived StO_2 _variables were calculated automatically by the InSpectra Analysis Program: the StO_2 _desaturation slope (desStO_2_, expressed as percentage per minute), the StO_2 _recovery slope (recStO_2_, expressed as percentage per second) and the hyperaemia recovery area (Figure 1).

### Experimental protocol

In all patients, fluid responsiveness was evaluated before induction of anaesthesia by use of a passive leg raising test as described previously [20,21]. In cases of passive leg raising-induced changes in SV >16%, a 250-mL fluid bolus of hydroxyethylstarch (HES 130/0.4 (Voluven); Fresenius-Kabi AG, Bad Homburg, Germany) was delivered over a period of 15 minutes before anaesthesia induction. After anaesthesia induction and a five-minute period of stabilisation (which was defined as the baseline state), the first set of measurements was recorded (systolic arterial pressure (PAS), diastolic arterial pressure (PAD) ultrasound, mean arterial pressure (PAM), CI, SV, baseline StO_2_, desStO_2_, recStO_2 _and hyperaemia recovery area). For each episode of suspected hypovolaemia, defined as PPV >13% and SVV >12% [5], VE was performed using a 500-mL bolus of hydroxyethylstarch delivered over a 15-minute period. Fluid responsiveness was defined as an increase in SV of ≥15% [3]. Before and after each VE, an additional set of measurements was obtained (PAS, PAD, PAM, CI, SV, baseline StO_2_, desStO_2_, recStO_2 _and hyperaemia recovery area).

### Statistical analysis

We subdivided the population into two groups based on the percentage increase in SV after intravascular VE: (1) positive response to fluid challenge when SV was ≥15% and (2) negative response to fluid challenge when SV was <15%. The results were tested for normality using the one-sample Kolmogorov-Smirnov goodness-of-fit test. Normally distributed data are presented as means ± standard deviation (SD) or and non-normally distributed data as medians with 25th and 75th percentiles. The χ^2 ^test was used to compare categorical data. Quantitative data were compared using analysis of variance (ANOVA) when the distributions were normal and the variances were equivalent; otherwise, they were compared using the Kruskal-Wallis H-test. The paired Student's *t*-test was used to compare data at two different time points, with adjustment of the *t*-statistics whenever indicated, to take into account the presence of several measurements in the patient studied. The within-group effect of fluid loading was analysed using ANOVA or the Kruskal-Wallis H-test as appropriate. To assess the reproducibility of VOT-derived StO_2 _variables, the coefficient of variability was calculated to obtain the StO_2 _recovery slopes for both positive and negative responses to fluid loading [22]. Differences between groups were assessed using Student's *t*-test or a Mann-Whitney *U *test as appropriate. A mixed model using the restricted maximum likelihood method was used to estimate covariance components, taking into account the random effects of patient and time (before and after fluid loading) and the covariate interaction group × time. The receiver operating characteristic (ROC) curve was also generated for the StO_2 _recovery slope, and area under the ROC curve, sensitivity, specificity, positive predictive value and negative predictive value were calculated for recStO_2_. When applicable, correlations were evaluated on the basis of the Spearman's ρ coefficient. Statistical analysis was performed using SEM version 2.0 software [23]. *P *< 0.05 was considered statistically significant.

## Results

Table 1 summarises the baseline demographic clinical characteristics of the 24 patients. On the basis of the passive leg-raising test, no patient was considered preload-dependent before induction of anaesthesia. The duration of the surgical procedures ranged from 75 to 300 minutes (median, 120 minutes). Table 2 shows the baseline macrocirculatory and thenar StO_2 _curve variables, which were recorded after induction of anaesthesia. No patients required vasopressor therapy during the study period.

**Table 1:** General characteristics of the study population before induction of anaesthesia^a^

Variables	Values
Age, years	62 ± 13
Sex ratio, % (M/F)	54/46
Height, cm	167 ± 8
Body surface area, m^2^	1.8 ± 0.2
BMI, kg/m^2^	24 ± 4
Comorbidities, %	
Hypertension	54
Ischaemic heart disease	4
Diabetes mellitus	17
COPD	13
Smokers	33
MAP, mmHg	93 ± 17
HR, beats/minute	70 ± 18
Hb, g/dL	13 ± 2
SpO_2_, %	97 ± 2
StO_2_, %	80 (77 to 83)

^a^BMI, body mass index; COPD, chronic obstructive pulmonary disease; Hb, haemoglobin; HR, heart rate; MAP, mean arterial pressure; SpO_2_, peripheral oxygen saturation; StO_2_, tissue oxygen saturation. Data are absolute values, means ± SD or medians (25th and 75th percentiles).

**Table 2:** Macrocirculatory and muscle tissue oxygen saturation curve variables at baseline after induction of anaesthesia^a^

Variables	Values
Systolic arterial pressure, mmHg	110 ± 16
Diastolic arterial pressure, mmHg	57 ± 10
Mean arterial pressure, mmHg	73 ± 12
Heart rate, beats/minute	71 ± 13
Stroke volume, mL	70 ± 20
Cardiac output, L/minute	5.0 ± 0.9
PPV, %	8 (5.75 to 8.25)
SVV, %	7 (6.0 to 8.0)
SpO_2_, %	98 ± 1
StO_2_, %	86 (78 to 88)
desStO_2_, %/minute	-10.4 (-10.2 to -8.8)
recStO_2_, %/second	5.1 (3.89 to 5.53)
Hyperaemia recovery area, AU	16.9 (13.1 to 20.6)

^a^PPV, pulse pressure variation; SVV, stroke volume variation; SpO_2_, peripheral oxygen saturation; StO_2_, tissue oxygen saturation; desStO_2_, StO_2 _desaturation slope; recStO_2_, StO_2 _recovery slope; AU, arbitrary units. Data are means ± SD or medians (25th and 75th percentiles).

### Macrocirculatory variables

Compared to baseline values, hypovolaemia was associated with a significant reduction in SV (70 ± 20 mL vs. 58 ± 12 mL; *P *= 0.038), but CO was not significantly different (5.0 ± 0.9 L/minute vs. 4.5 ± 0.9 L/minute; *P *= 0.13). Before VE, PPV was 16% (range, 14% to 18%) and SVV was 15% (range, 14% to 16%). A total of 42 fluid challenges (one to three per patient) were performed. In the whole study population, delivery of a fluid bolus was associated with a 27.7 ± 20% increase in SV (*P *< 0.001). According to the expected 15% increase in SV, 29 (69%) of 42 fluid challenges were positive in relation to VE (mean change in SV: 36 ± 17%) and 13 fluid challenges were negative (mean change in SV: 10 ± 16%). There was a statistically significant difference (*P *< 0.001) in SV changes between positive and negative responses to fluid challenge. With regard to the type of fluid response, there was a statistically significant difference in PPV and SVV values before VE for the positive and negative fluid responses [PPV: 16% (15% to 18%) vs. 14% (13% to 15%), *P *= 0.001, and SVV: 14% (13% to 16%) vs. 16% (15% to 16%), *P *= 0.03, for positive and negative fluid responses, respectively]. In the positive fluid challenge, VE also induced significant changes in CO and MAP, but there were no evident differences induced by the negative fluid challenge (Table 3). There was no statistically significant difference in haemoglobin levels during VE (12.6 ± 2 g/dL before VE vs. 11.7 ± 2 g/dL after VE; *P *= 0.10).

**Table 3:** Changes in macrocirculatory and microcirculatory variables during fluid challenge^a^

	Positive fluid challenge (*n *= 29)		Negative fluid challenge (*n *= 13)	
Variables	Before VE	After VE	Before VE	After VE
SAP, mmHg	94 ± 15	112 ± 15†	104 ± 20	106 ± 11
DAP, mmHg	53 ± 10	57 ± 9	52 ± 9	51 ± 7
MAP, mmHg	66 ± 11	75 ± 10†	69 ± 12	70 ± 7
HR, beats/minute	77 ± 16	75 ± 14	78 ± 13	75 ± 12
SV, mL	61 ± 12	83 ± 19†	64 ± 15	69 ± 14
CO, L/minute	4.7 ± 1.2	6.3 ± 1.7†	4.9 ± 1.5	5.2 ± 1.3
SVV, %	14 (13 to 16)	6 (5 to 7)†	16 (15 to 16)	6 (5 to 8)†
PPV, %	16 (15 to 18)	5 (3 to 6)†	14 (13 to 15)	5 (4 to 8)†
Pre-VOT StO_2_, %	84 (77 to 91)	88 (79 - 93)	83 (77 to 90)	84 (79 to 91)
desStO_2_, %/minute	-10.4 (-12.2 to -9.2)	-10.9 (-10.7 to -6.5)	-9.9 (-10.7 to -9.7)	-10.8 (-13.1 to -9.9)
Hyperaemia recovery area, AU	12.6 (7.5 to 21.5)	12.2 (7.3 to 22.9)	12.9 (10.7 to 20.9)	12.8 (8.9 to 19.8)

^a^CO, cardiac output; DAP, diastolic arterial pressure; HR, heart rate; MAP, mean arterial pressure; SAP, systolic arterial pressure; StO_2_, tissue oxygen saturation; desStO_2_, StO_2 _desaturation slope; recStO_2_, StO_2 _recovery slope; PPV, pulse pressure variation; SVV, stroke volume variation; SV, stroke volume; VE, volume expansion; VOT, vascular occlusion test; AU, arbitrary units. Data are means ± SD or medians (25th and 75th percentiles). †*P *< 0.05 after vs. before volume expansion.

### Near-infrared spectroscopy variables

There was no significant difference in the mean StO_2 _values before and after induction of anaesthesia [80% (77% to 83%) vs. 86% (78% to 88%); *P *= 0.15]. Table 3 shows the mean values of the NIRS variables before and after VE. Before VE there was no significant difference in pre-VOT StO_2 _values during hypovolaemia relative to baseline [86% (78% to 88%) vs. 84% (77% to 91%); *P *= 0.83] and no significant difference between positive and negative fluid challenges (*P *= 0.36). There was also no significant difference in desStO_2 _values before VE relative to baseline (*P *= 0.53) or between positive and negative fluid challenges (Table 3). Hypovolaemia was associated with a significant reduction in recStO_2 _values relative to baseline [5.1% (3.89% to 5.53%)/second vs. 3.57% (2.71% to 4.58%)/second; *P *= 0.004]. There was also a significant difference in recStO_2 _before VE in the positive and negative fluid challenges (*P *= 0.02) (Table 3). Before VE there was no significant difference in StO_2 _hyperaemia recovery area relative to baseline (*P *= 0.30) or between positive and negative fluid challenges (*P *= 0.29) (Table 3).

**Figure 2:**
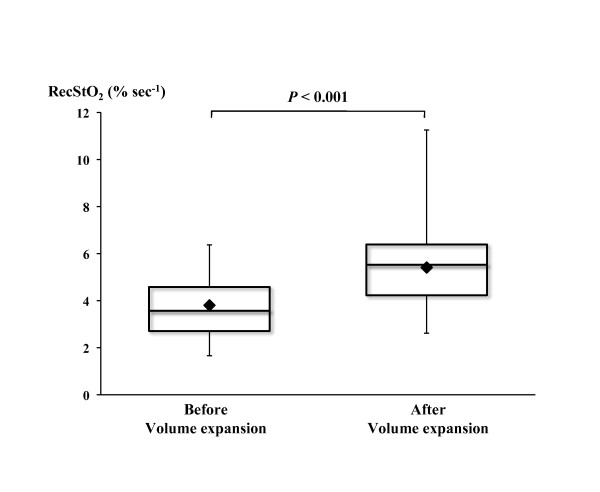
**Changes in the tissue oxygen saturation recovery slope during fluid challenge**. Boxplots showing the median (horizontal lines within the boxes) 75th and 25th percentiles (upper and lower edges of the boxes), maximum and minimum values (upper and lower bars), and means (dark diamonds within the boxes). RecStO_2_, StO_2 _recovery slope; StO_2_, tissue oxygen saturation.

**Figure 3:**
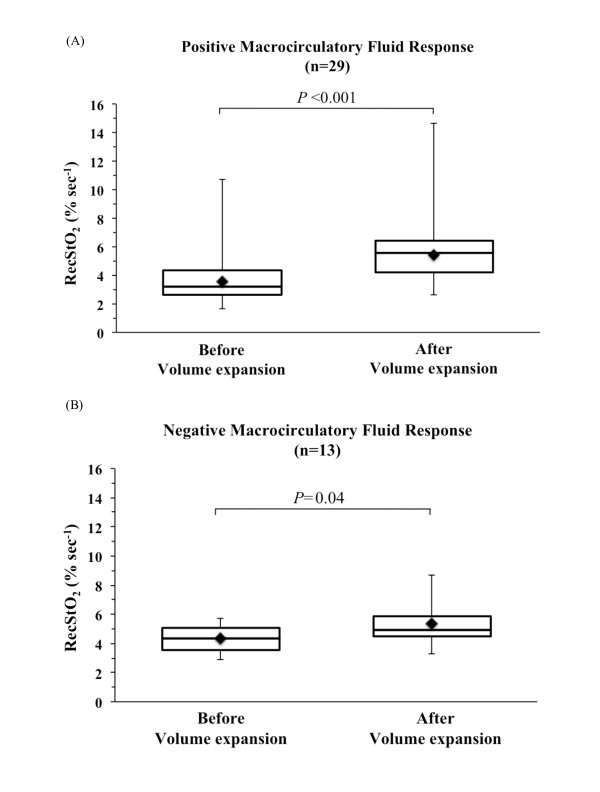
**Changes in the thenar tissue oxygen saturation recovery slope before and after volume expansion for positive (A) and negative (B) fluid events**. A positive fluid event was characterised by a 15% increase in stroke volume with pulse pressure variation >13% and stroke volume variation >12%. Boxplots show the medians (horizontal lines within the boxes) with 75th and 25th percentiles (upper and lower edges of the boxes), maximum and minimum values (upper and lower bars) and means (dark diamonds within the boxes). RecStO_2_, tissue oxygen saturation recovery slope.

There was no significant change in pre-VOT StO_2 _after VE (*P *= 0.22) in the positive and negative fluid challenges (Table 3). VE resulted in an overall increase in recStO_2 _of 50 ± 47% (*P *< 0.001) (Figure 2). For positive fluid challenge, the VE-induced increase in recStO_2 _was 62 ± 49% (*P *< 0.001); for negative fluid challenge, the VE-induced increase in recStO_2 _was 26 ± 34% (*P *= 0.04) (Figure 3). There was a significant difference (*P *= 0.016) in VE-induced increase in recStO_2 _between positive and negative responses to VE. The coefficient of variability of recStO_2 _was 25% and 33% for the negative fluid challenge group and the positive fluid challenge group, respectively, with a statistically significant difference between groups (*P *= 0.0453). The results obtained with the mixed model showed a significant interaction between covariates (*P *= 0.039). Variance attributable to each random effect was 45.27% for patient, 49.60% for time and 5.13% for residual variability. The area under the ROC curve of recStO_2 _was 0.740 (95% confidence interval, 0.56 to 0.91). The optimal cutoff value for recStO_2 _was 18% (sensitivity 85.7%, specificity 61.5%, positive predictive value 82.8% and negative predictive value 66.7%). There were no significant changes in desStO_2 _or StO_2 _hyperaemia area during VE and no differences in positive and negative fluid challenges (Table 3).

### Changes in stroke volume, cardiac output and tissue oxygen saturation recovery slope during volume expansion

**Figure 4:**
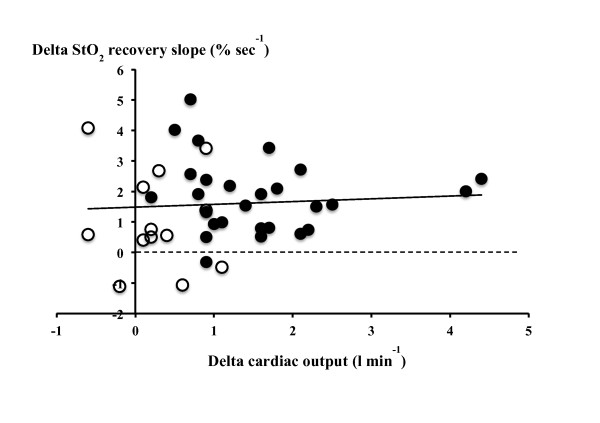
**Relationship between changes in cardiac output and changes in the tissue oxygen saturation recovery slope in all fluid challenges**. *x*-axis: Difference in cardiac output after and before the fluid challenge (L/minute). *y*-axis: Difference in the tissue oxygen saturation (StO_2_) recovery slope after and before the fluid challenge (percentage per second). Closed circles represent positive fluid challenges. Open circles represent negative fluid challenges. Spearman's ρ coefficient = 0.10, *P *= 0.56.

No significant relationship was observed between VE-induced changes in SV and changes in the StO_2 _recovery slope (Spearman's ρ coefficient = 0.15, *P *= 0.33) or between VE-induced changes in CO and the StO_2 _recovery slope (Figure 4).

## Discussion

The main findings of our study are that (1) hypovolaemia is associated with significant alterations in NIRS variables measured at the thenar eminence and (2) restoring effective intravascular volume with fluid loading significantly improves the StO_2 _recovery slope. Furthermore, during apparently ineffective fluid loading (that is, without significant changes in systemic haemodynamics), VE also leads to a significant improvement in the StO_2 _recovery slope.

Monitoring the effects of fluid therapy at the bedside remains a cornerstone in the operating room and ICU. Although there are limitations [24,25], previous research indicates that dynamic indices (based on flow or preload parameters) are useful for predicting individualised fluid responsiveness [26] and prevention of excessive fluid intake. However, preload responsiveness does not equate to fluid requirement [27]. We used a PPV value >13% and a SVV value >12% to predict fluid responsiveness during mechanical ventilation with a tidal volume >8 mL/kg [5], but 69% of the significant changes in SV were induced by VE and increased CO. Conversely, one-third of the volume challenges were not accompanied by a significant increase in SV, although the patients were supposed to be on the steep portion of the Frank-Starling curve. This was very close to the range that was previously reported (40% to 72%) [28]. We used a fixed cutoff value for SV (≥15% increase) to dichotomise positive and negative responses to fluid loads [3]. Nevertheless, because the response to a given volume load is a continuum ranging from no increase to a large increase in SV, it can be assumed that a lower threshold value (10% instead of 15%) would have led to a different level of significance. In addition, although we used generation software whose accuracy has been well established to estimate CO in surgical patients, concerns have been raised regarding its reliability to track changes in SV or CO in some patients [29,30]. Taken together, this information could explain, at least in part, the absence of significant changes in some measurements of CO despite reductions in both PPV and SVV values with fluid loading. Nevertheless, fluid resuscitation is designed to restore systemic haemodynamics, so determining whether a patient is preload-dependent provides only part of the answer, because fluid challenge should be performed within the context of known or suspected tissue hypoperfusion [27].

In our study, preload responsiveness was associated with a 25% decrease in the StO_2 _recovery slope. Previous data have suggested that hypovolaemia leads to inadequate perfusion of the microcirculation, which results in insufficient oxygen availability [31]. The StO_2 _recovery slope is believed to reflect the microvascular blood flow response to a transient tissue hypoxia-induced oxygen deficit created by the ischaemic stimulus [19]. Vasodilatation of arterioles and recruitment of closed capillaries are responsible for this reactive hyperaemia [16]. We used a fixed StO_2 _target of 40%, as recommended by other investigators [32,33], and found that the baseline StO_2 _recovery slopes were very close to those previously reported in healthy volunteers [15,33]. Previous studies have suggested that StO_2 _values can be used to detect changes in peripheral tissue oxygenation resulting from a lower-body negative pressure model of simulated central hypovolaemia [34]. A reduction in muscle oxygen was also found to be an earlier indicator of hypovolaemia than the standard clinical measures (heart rate and blood pressure) [35]. Nevertheless, previous studies have suggested that resting StO_2 _values are insensitive to the assessment of tissue hypoperfusion [36]. Our results, which indicate similar resting StO_2 _values in spite of an insufficient flow, support the findings of these previous studies.

We also found a 50% increase in the StO_2 _recovery slope with fluid loading. This suggests that restoration of intravascular volume in preload-dependent patients improves muscle tissue oxygenation and increases SV and CO. This finding may have important clinical implications, because the ultimate goal of resuscitation should be improvement of tissue oxygenation and perfusion. We hypothesise that this is due to improved microvessel recruitment during the fluid challenge, together with an increase venular blood compartment volume, despite the absence of macrocirculatory changes. It is unlikely that changes in the StO_2 _recovery slope with VE were due to changes in rheologic factors, because we found no significant differences in haemoglobin levels before and after VE. In addition, Creteur *et al*. [37] recently performed VOT before and after red blood cell transfusion and reported no differences despite the different haemoglobin levels. It must be stressed that the StO_2 _recovery slope remained low, or even decreased, in some of our patients after VE (Figure 2), even though CO improved with VE. This is in agreement with the hypothesis that VE can cause apparent improvement in systemic parameters, even though microcirculation and tissue oxygenation remain uncorrected. In addition, a 500-mL fluid infusion might have been insufficient in some patients.

Our study has several limitations that need to be addressed. First, we studied only surgical patients. Although major surgery is associated with significant impairment in both microvascular flow and tissue oxygenation [38], our data should not be extrapolated to other, more specific patient populations (with an increased intersubject variability) until further investigations are carried out. In addition, repeated measurements were performed in some patients. An advantage of analysing the response to repeated fluid loading is that it reproduces daily practice, when fluid responsiveness has to be evaluated in the same patient on different occasions. Second, we did not evaluate patient outcomes. In other words, we did not determine whether higher StO_2 _recovery slopes were associated reduced organ failure. Third, we placed the NIRS probe on the thenar eminence, a region with very little fat, and therefore there was little interference with the spectroscopic measurements. Although measurement at this site have very low variance [39] and even though we used a standardised VOT, the interpretation of VOT should be viewed with caution. Fourth, in all patients, anaesthesia was maintained by continuous infusion of propofol, which previous research has indicated increases blood flow to the muscles and the vascular bed [40]. Although all the VOTs were performed while patients were under similar conditions of anaesthesia, we cannot exclude the possibility that our results would have been different if different drugs had been used. Fifth, none of the patients were given vasoactive drugs during the study protocol. Although norepinephrine can improve the StO_2 _recovery slope [16], the effects of VE in such conditions has not been evaluated.

In conclusion, with respect to our study conditions, we found that preload dependence is associated with significant changes in the StO_2 _recovery slope based on NIRS measurements at the thenar eminence. In addition, our findings suggest that VE can improve tissue oxygenation. Taken together, our results suggest that measurements of StO_2 _using a standardised VOT could be a useful complementary tool along with the dynamic indices to improve fluid optimisation. Further studies are warranted to validate this hypothesis.

## Key messages

• When hypovolaemia is suspected, fluid loading should restore systemic haemodynamics, but the ultimate goal of fluid resuscitation should be improvement of tissue perfusion and oxygenation.

• Preload dependence is associated with significant alterations in the StO_2 _recovery slope based on NIRS measurements, suggesting the coexistence of microcirculatory abnormalities.

• VE induces positive effects at the level of muscle tissue oxygenation as measured by the elevation of the StO_2 _recovery slope.

• The StO_2 _recovery slope better assesses the effects of hypovolaemia and fluid loading on muscle tissue oxygenation than other NIRS-derived variables.

## Abbreviations

CI: cardiac index; CO: cardiac output; desStO_2_: StO_2 _desaturation slope; NIRS: near-infrared spectroscopy; PPV: pulse pressure variation; recStO_2_: StO_2 _recovery slope; StO_2_: tissue oxygen saturation; SV: stroke volume; SVV: stroke volume variation; VE: volume expansion.

## Competing interests

The authors declare that they have no competing interests.

## Authors' contributions

EF and ER conceived and designed the original study. SC and AP were responsible for patient enrolment and participated in data acquisition. EF, ER, BV, JD and JEB drafted the manuscript. All authors read and approved the final manuscript.
